# Persistent atrial fibrillation over 3 years is associated with higher recurrence after catheter ablation

**DOI:** 10.1111/jce.14345

**Published:** 2020-01-15

**Authors:** Hee Tae Yu, In‐Soo Kim, Tae‐Hoon Kim, Jae‐Sun Uhm, Jong‐Youn Kim, Boyoung Joung, Moon‐Hyoung Lee, Hui‐Nam Pak

**Affiliations:** ^1^ Division of Cardiology Yonsei University Health System Seoul Republic of Korea

**Keywords:** atrial fibrillation, catheter ablation, duration, persistent, recurrence

## Abstract

**Instruction:**

Longer atrial fibrillation (AF) durations have higher recurrence rates after rhythm control. However, there is limited data on the effect of the AF duration on recurrence after atrial fibrillation catheter ablation (AFCA). In the present study, we investigated the rhythm outcome of AFCA according to the AF duration based on the first electrocardiogram (ECG) diagnosis.

**Methods and Results:**

We included 1005 patients with AF (75% male, 59 ± 11 years old) who underwent AFCA and whose first ECG diagnosis time point was evident. The clinical characteristics and rhythm outcomes were compared based on the AF duration (≤3 years, n = 537; >3 years, n = 468) and AF burden (paroxysmal atrial fibrillation [PAF], n = 387; persistent atrial fibrillation [PeAF], n = 618). Longer AF durations were associated with older age (*P* = .020), larger left atrial size (*P* = .009) and a higher number of patients with hypertension (*P* < .001) or PeAF (*P* < .001). During 24 ± 22 months of follow‐up, the postablation clinical recurrence rate was higher in patients with a longer AF duration (logrank *P* = .002). The AF recurrence rate was significantly higher in PeAF patients with an AF duration >3 years (logrank *P* = 0.009), but not in subjects with PAF (logrank *P* = .939). In a multivariate Cox regression analysis, a longer AF duration was significantly associated with a higher clinical recurrence rate after AFCA in PeAF patients (adjusted hazard ratio, 1.06; range, 1.03‐0.10; *P* = 0.001), but not PAF.

**Conclusion:**

Although longer AF duration was associated with higher clinical recurrence rates after AFCA, the rate was significant in patients with PeAF lasting >3 years, but not in PAF patients.

## 1 INTRODUCTION

Atrial fibrillation (AF) is a chronic degenerative disease with a 1.5% prevalence of the total population and continuously increasing in the elderly population.^1^ AF is also a progressive disease with annual progression rates of 7% to 15%,^2^, ^3^ and more than 50% of paroxysmal atrial fibrillation (PAF) will progress to persistent atrial fibrillation (PeAF) within 10 years.^4^ AF progresses more rapidly to more persistent forms in PeAF than in PAF patients,^5^ and in patients with a higher number of cardiovascular risk factors.^6^ The aggressive rhythm control slows the AF progression, and the AF progression rate is 3.3‐fold lower in the rhythm‐control group than the rate control group.^3^, ^6^ Catheter ablation of AF is an effective rhythm control strategy that reduces the AF burden, heart failure mortality,^7^ risks of a stroke,^8^ and risk of cognitive dysfunction.^9^

The AF duration has been considered an important prognostic factor for the rhythm outcome after a maze procedure^10^ or AF ablation.^11^ However, AF is less consistent with symptoms and asymptomatic subclinical AF is common. Reportedly, over 40% of patients did not complain of any significant symptoms under an appropriate rate control.^12^ Therefore, determining the exact AF duration based on the symptoms is difficult. However, we measured the time period from the first electrocardiogram (ECG) diagnosis of AF to the de novo atrial fibrillation catheter ablation (AFCA) in our AF ablation study population. In addition, we monitored the postablation AF recurrence by a consistent long‐term rhythm monitoring protocol according to the 2012 HRS/EHRA/ECAS Expert consensus statement guidelines.^13^, ^14^ In the present study, we tested the hypothesis whether the documented ECG‐based AF duration was closely associated with a high recurrence rate after radiofrequency AFCA.

## 2 METHODS

### 2.1 Study population

**Figure 1:**
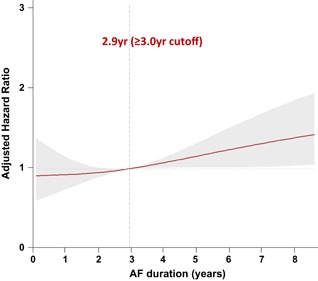
The adjusted hazard ratio of atrial fibrillation (AF) duration for AF recurrence. Adjusted for age, sex, body mass index, paroxysmal atrial fibrillation, heart failure, hypertension, diabetes, stroke or transient ischemic attack, left atrium diameter, left ventricle ejection fraction

The study protocol adhered to the Declaration of Helsinki and was approved by the Institutional Review Board of the Yonsei University Health System. All patients provided written informed consent for inclusion in the Yonsei AF Ablation Registry Database (registered at clinicaltrials.gov as NCT02138695). From March 2009 to September 2017, 1005 patients who underwent AFCA for AF and whose first ECG diagnosis time point was evident were analyzed (75% male, 59 ± 11 years of age, 39% PAF). AF onset was defined as the index ECG showing AF. AF that terminates spontaneously or with intervention within 7 days of onset was classified as PAF.^15^ We analyzed the sensitivity and specificity for predicting clinical recurrences of AF after AFCA on the basis of the different cut‐off ranges of the AF duration (Figure 1 and Table S1). As a result of the analysis, the 3‐year cut‐off value represented the best dichotomy among a number of tested cut‐off values. Therefore, we compared the patients with an AF duration of less than 3 years and those with that longer than 3 years based on a preliminary adjusted hazard ratio (HR) analysis. The clinical characteristics and rhythm outcomes were compared based on the AF duration (≤3 years, n = 537; >3 years, n = 468) and AF burden (PAF, n = 387; PeAF, n = 618). Exclusion criteria were as follows: (a) valvular AF, (b) structural heart disease other than left ventricular hypertrophy, (c) left atrial (LA) diameter ≥60 mm, and (d) a history of a previous AF ablation or cardiac surgery. All antiarrhythmic drugs (AADs) were discontinued for a minimum period of five half‐lives before the procedure. Anticoagulation therapy was maintained before the catheter ablation. The anatomy of the LA and the pulmonary veins (PVs) in all patients was imaged using three‐dimensional (3D) spiral computed tomography (CT) scans (64 Channel, LightSpeed Volume CT, Philips, Brilliance 63, Amsterdam, Netherlands).

### 2.2 Electroanatomical mapping and echocardiographic evaluation

Intracardiac electrograms were recorded using the Prucka CardioLab Electrophysiology System (General Electric Medical Systems, Inc, Milwaukee, WI), and AFCA was performed in all patients using 3D electroanatomical mapping (NavX, St Jude Medical, Inc, Minnetonka, MN) merged with 3D spiral CT. Transseptal punctures were made and multiview pulmonary venograms were obtained. Systemic anticoagulation was performed with intravenous heparin to maintain an activated clotting time of 350 to 400 seconds during the procedure. For electroanatomical mapping, the 3D geometry of both the LA and the PVs was generated using the NavX system and then merged with 3D spiral CT images. LA electrogram voltage maps were generated during high right atrial pacing at 500 ms to prevent rate‐dependent activation changes after maintaining sinus rhythm by circumferential pulmonary vein isolation (CPVI) with or without cardioversion. If frequently recurring AF persisted after three attempts at cardioversion, no further efforts were made to generate an LA voltage map. The peak‐to‐peak amplitude of the contact bipolar electrograms was obtained from 350 to 500 points on the LA endocardium and the mean LA electrogram voltage was calculated.

All patients underwent transthoracic echocardiography (Sonos 5500; Philips Medical Systems, Andover, MA; Vivid 7, GE Vingmed Ultrasound, Horten, Norway) before the AFCA. Transesophageal echocardiography or intracardiac echocardiography was performed to exclude any intracardiac thrombi. The emptying velocity of the LA appendage was measured in all patients.

### 2.3 Atrial fibrillation catheter ablation

The details of the AFCA technique and strategy were described in our previous study.^16^ Briefly, for the CPVI ablation, continuous circumferential lesions were created at the level of the LA antrum encircling the right and left PVs guided by the NavX system using an open‐irrigated, 3.5‐mm tip deflectable catheter at 30 to 35 W and 45°C (ThermoCool, Johnson & Johnson, Inc, Diamond Bar, CA; Cool Flex, St Jude Medical, Inc). A CPVI and cavotricuspid isthmus ablation were performed in all patients. The endpoints of both the CPVI and cavotricuspid isthmus block were defined based on bidirectional pacing. For patients with persistent AF, a roofline, posterior‐inferior line, and anterior line were added as a standard lesion set. At the operator's discretion, additional ablation of the superior vena cava, non‐PV foci, or complex fractionated electrograms was conducted. The procedure ended when no immediate recurrence of AF was observed within 10 minutes after cardioversion with an isoproterenol infusion (5‐10 μg/min). Non‐PV foci under an isoproterenol infusion were also ablated.

### 2.4 Postablation follow‐up

The patients without antiarrhythmic medications were discharged after the procedure unless early recurrence of AF/AT or symptomatic frequent atrial premature beats were evident. Patients visited the outpatient clinic regularly at 1, 3, 6, and 12 months and then every 6 months or whenever symptoms occurred after the AFCA. All patients underwent ECG at each visit and 24‐hour Holter recordings at 3 and 6 months and every 6 months thereafter following the 2012 HRS/EHRA/ECAS Expert Consensus Statement guidelines.^13^ Holter monitoring or event monitor recordings were obtained when patients reported symptoms of palpitation suggestive of arrhythmia recurrence. AF recurrence was defined as any episode of AF or AT of at least 30 seconds in duration. Any ECG documentation of an AF recurrence within a 3‐month blanking period was diagnosed as an early recurrence and an AF recurrence more than 3 months after the procedure was diagnosed as a clinical recurrence.

### 2.5 Statistical analyses

Continuous variables were summarized as the mean ± standard deviation and compared using a Student *t* test and analysis of variance. Categorical variables were summarized as a percentage of the group total and compared using the *χ* ^2^ test or Fisher's exact test. To predict clinical recurrence of AF after AFCA on the basis of different cut‐off ranges of AF duration, receiver operating characteristic curves were constructed. A multivariate Cox regression analysis was used to identify the predictors of clinical recurrence. Variables with a *P* value less than .05 based on univariate analysis were selected for the multivariate analysis. In addition, if a significant correlation between the selected variables (*R* > .5) was observed, only one variable was used to avoid multicollinearity in the multivariate regression analysis. A Kaplan‐Meier analysis with a logrank test was used to calculate the AF recurrence‐free survival over time and to compare the recurrence rates across the groups. A *P* value less than .05 was considered statistically significant. The statistical analyses were performed using SPSS (version 25.0; Statistical Package for Social Sciences, Chicago, IL) software for Windows.

## 3 RESULTS

### 3.1 Baseline characteristics

The baseline clinical characteristics of the study population are shown in Table 1. We compared the patient with AF duration less than 3 years and those with longer than 3 years based on preliminary adjusted HR analysis (Figure 1). The sex distribution did not statistically significantly differ between the two groups. A longer AF duration was associated with older age (*P* = .020), a higher number of patients with hypertension (*P* < .001) and PeAF (*P* < .001). The LA diameter on echocardiography also increased as the AF duration increased (*P* = .009), while the endocardial voltage of the LA decreased as the AF duration increased (*P* < .001). Other comorbidities including the CHA_2_DS_2_‐VASc score and baseline echocardiographic and CT parameters did not significantly differ between the groups.

**Table 1:** Baseline characteristics of the patients

	AF duration ≤3 y (n = 537)	AF duration >3 y (n = 468)	*P* value
Age, y	58.2 ± 11.2	59.7 ± 10.0	.020*
Male sex, n (%)	391 (72.8)	363 (77.6)	.083
Paroxysmal AF, n (%)	238 (44.3)	149 (31.8)	<.001*
BSA, m^2^	1.81 ± 0.19	1.82 ± 0.18	.795
BMI, kg/m^2^	25.1 ± 3.1	25.0 ± 3.4	.529
Comorbidities			
Heart failure, n (%)	75 (14.0)	66 (14.1)	.951
Hypertension, n (%)	211 (39.3)	237 (50.6)	<.001*
Diabetes mellitus, n (%)	88 (16.4)	73 (15.6)	.745
Stroke or TIA, n (%)	84 (15.6)	64 (13.7)	.380
Vascular Disease, n (%)	74 (13.8)	69 (14.7)	.663
CHA_2_DS_2_‐VASc score	1.8 ± 1.6	1.8 ± 1.5	.567
Echocardiography			
LA diameter, mm	42.1 ± 6.1	43.2 ± 6.3	.009*
LA volume index, mL/m^2^	39.2 ± 13.3	40.7 ± 13.0	.077
LV ejection fraction, %	62.5 ± 8.3	61.9 ± 9.3	.271
E/Em	10.3 ± 4.6	10.4 ± 4.0	.865
LVEDD, mm	49.9 ± 4.8	50.2 ± 4.6	.264
LAA emptying velocity, cm/s	44.8 ± 21.7	43.1 ± 20.6	.314
CT/NavX (n = 976)			
LA volume/BSA, mL/m^2^	87.7 ± 24.1	91.3 ± 26.0	.025*
Pericardial fat volume, cm^3^	128.7 ± 58.9	128.9 ± 56.0	.956
LA endocardial voltage, mV	1.28 ± 0.64	1.04 ± 0.55	<.001*

Abbreviations: AF, atrial fibrillation; BMI, body mass index; BP, blood pressure; BSA, body surface area; CT, computed tomography; E/Em, early mitral inflow velocity over the early diastolic mitral annular velocity; LA, left atrium; LAA, left atrial appendage; LV, left ventricle; LVEDD, left ventricular end‐diastolic dimension; TIA, transient ischemic attack.

* *P* < .05.

### 3.2 Procedural results and clinical outcome after catheter ablation of AF

**Figure 2:**
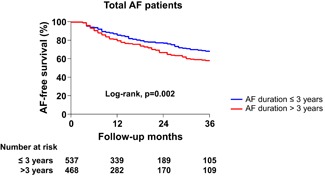
Kaplan‐Meier analysis of the AF recurrence‐free survival after catheter ablation in the total study population. AF, atrial fibrillation

**Figure 3:**
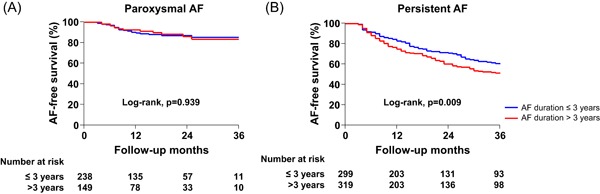
Kaplan‐Meier analysis of the AF recurrence‐free survival after catheter ablation in patients with paroxysmal AF (A) and persistent AF (B). AF, atrial fibrillation

The procedural results and clinical outcomes are summarized in Table 2. The total procedure time (*P* = .152) and ablation time (*P* = .548) did not statistically differ between the two groups, however, the AAD utility rates at discharge were higher in the patients with a longer preprocedural AF duration (14.2% vs 21.0%; *P* = .005). During 24 ± 22 months of follow‐up after the AF ablation, the early recurrence rate (27.6% vs 39.0%; *P* < .001) and clinical recurrence rate (24.9% vs 36.9%; *P* < .001) were significantly higher in the longer AF duration group (Table 2). The Kaplan‐Meier analysis also showed a significantly higher clinical recurrence of AF in patients with a longer preprocedural AF duration (Figure 2, logrank *P* = .002). In the subgroup analysis based on the AF type, the clinical recurrence rate did not significantly differ among the patients with PAF (Figure 3A, logrank *P* = .939). However, in the PeAF group, the clinical recurrence rate was significantly higher in patients with a longer AF duration (Figure 3B, logrank *P* = .009).

**Table 2:** Procedural results and clinical outcomes

	AF duration ≤3 y (n = 537)	AF duration >3 y (n = 468)	*P* value
Procedure time, min	195.2 ± 48.4	200.0 ± 56.5	.152
Ablation time, s	5275 ± 1656	5204 ± 2009	.548
AAD at discharge, n (%)	76 (14.2)	98 (21.0)	.005*
Follow‐up duration, mo	23.0 ± 20.9	24.2 ± 22.9	.363
Early recurrence, n (%)	141 (27.6)	174 (39.0)	<.001*
Clinical recurrence, n (%)	127 (24.9)	164 (36.9)	<.001*

Abbreviations: AAD, antiarrhythmic drug; AF, atrial fibrillation.

* *P* < .05.

### 3.3 AF duration as a predictor of a clinical recurrence of AF after catheter ablation

Univariate and multivariate Cox regression analyses were performed to identify the predictors of a clinical recurrence of AF after catheter ablation (Table 3). In patients with PAF, a female sex (HR, 0.470; 95% confidence interval [CI], 0.233‐0.948; *P* = .035), a lower body surface area (HR, 0.131; 95% CI, 0.019‐0.913; *P* = .040), and a lower LA voltage (HR, 0.386; 95% CI, 0.173‐0.862; *P* = .020) were associated with a higher clinical recurrence of AF after catheter ablation based on the univariate analysis. The AF duration was not significantly associated with a clinical recurrence after AFCA in patients with PAF. On the other hands, in patients with PeAF, a larger LA diameter (HR, 1.042; 95% CI, 1.012‐1.073; *P* = .005), a lower LA voltage (HR, 0.331; 95% CI, 0.213‐0.514; *P* < .001), and a longer AF duration (HR, 1.065; 95% CI, 1.033‐1.099; *P* < .001) were associated with a higher clinical recurrence of AF after catheter ablation based on the univariate analysis. In the multivariate Cox regression analysis, a larger LA diameter (adjusted HR, 1.058; 95% CI, 1.015‐1.102; *P* = .007), a lower LA voltage (adjusted HR, 0.365; 95% CI, 0.227‐0.586; *P* < .001) and a longer AF duration (adjusted HR, 1.064; 95% CI, 1.025‐1.104; *P* = .001) was significantly associated with a higher clinical recurrence of AF in patients with PeAF.

**Table 3:** Predictors for a clinical recurrence after catheter ablation of AF

	Univariate analysis		Multivariate analysis	
	HR (95% CI)	*P* value	HR (95% CI)	*P* value
Paroxysmal AF (n = 387)				
Age, y	0.989 (0.959‐1.021)	.499	0.970 (0.935‐1.008)	.117
Male sex	0.470 (0.233‐0.948)	.035*	0.537 (0.172‐1.677)	.284
BSA, m^2^	0.131 (0.019‐0.913)	.040*	0.402 (0.015‐10.490)	.584
BMI, kg/m^2^	0.940 (0.836‐1.056)	.295		
Heart failure	0.722 (0.211‐2.475)	.605		
Hypertension	1.294 (0.646‐2.593)	.467		
Diabetes mellitus	0.696 (0.236‐2.057)	.513		
Stroke or TIA	2.106 (0.929‐4.775)	.075		
Vascular disease	1.298 (0.510‐3.300)	.584		
LA diameter, mm	0.958 (0.899‐1.021)	.187		
LAVI, mL/m^2^	1.003 (0.975‐1.032)	.820		
LV ejection fraction (%)	1.014 (0.970‐1.060)	.544		
E/Em	0.939 (0.845‐1.043)	.238		
LA voltage, mV	0.386 (0.173‐0.862)	.020*	0.455 (0.191‐1.086)	.076
AF duration, y	0.923 (0.815‐1.046)	.208	0.983 (0.870‐1.110)	.781
Persistent AF (n = 618)				
Age, y	0.996 (0.981‐1.011)	.588	0.990 (0.971‐1.010)	.336
Male sex	1.063 (0.721‐1.569)	.757	1.047 (0.626‐1.752)	.860
BSA, m^2^	1.364 (0.569‐3.267)	.486		
BMI, kg/m^2^	1.008 (0.961‐1.058)	.737		
Heart failure	0.519 (0.325‐0.829)	.006*	0.429 (0.230‐0.800)	.008*
Hypertension	1.029 (0.744‐1.423)	.863		
Diabetes mellitus	0.729 (0.469‐1.133)	.160		
Stroke or TIA	0.788 (0.497‐1.250)	.312		
Vascular disease	1.034 (0.658‐1.625)	.886		
LA diameter, mm	1.042 (1.012‐1.073)	.005*	1.058 (1.015‐1.102)	.007*
LAVI, mL/m^2^	1.008 (0.995‐1.021)	.234		
LV ejection fraction (%)	1.009 (0.991‐1.029)	.322		
E/Em	0.976 (0.939‐1.014)	.207		
LA voltage, mV	0.331 (0.213‐0.514)	<.001*	0.365 (0.227‐0.586)	<.001*
AF duration, y	1.065 (1.033‐1.099)	<.001*	1.064 (1.025‐1.104)	.001*

Abbreviations: AF, atrial fibrillation; BMI, body mass index; BSA, body surface area; CI, confidence interval; E/Em, early mitral inflow velocity over the early diastolic mitral annular velocity; HR, hazard ratio; LA, left atrium; LAVI, left atrial volume index; LV, left ventricle; TIA, transient ischemic attack.

* *P* < .05.

**Figure 4:**
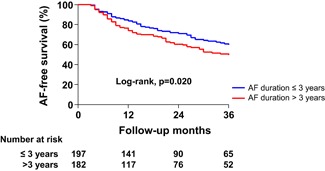
Kaplan‐Meier analysis of the AF recurrence‐free survival after catheter ablation in patients with persistent AF after a propensity score‐matching between patients with persistent and paroxysmal AF. AF, atrial fibrillation

Given the higher comorbidity of patients with PeAF, we additionally performed a propensity score match analysis between patients with PeAF and PAF in terms of age, sex, and the comorbidities that make up the CHA_2_DS_2_‐VASc score. After a propensity score matching, the matched population showed results consistent with the overall population in terms of the AF burden (paroxysmal or persistent) and AF duration (Table S2 and Figure 4).

## 4 DISCUSSION

### 4.1 Main findings

In the present study, the time period from the first ECG diagnosis of AF to the AFCA and clinical recurrence was measured using guideline‐based rhythm monitoring. The longer duration of AF was associated with a higher clinical recurrence rate after catheter ablation but was only significant in the patients with PeAF lasting more than 3 years. An AF duration‐dependent difference in the clinical recurrence rate was not observed in the patients with PAF.

### 4.2 AF progression and catheter ablation

AF is a chronic degenerative rhythm disorder with continuous progression associated with aging, LA enlargement, and heart failure.^17^ AF progression is more significant and accelerated in patients with associated hypertension, valvular disease, chronic lung disease, and a previous ischemic stroke.^18^, ^19^ AFCA is an effective rhythm control strategy to reduce the AF burden, however, the evolution of the abnormal atrial substrate progresses despite a successful ablation procedure and likewise the aging process.^20^ Furthermore, the development and advances in the catheter ablation technology, as well as the accumulated experience, have not been translated into a significant procedural success.^21^ Park et al^14^ found that extra‐PV ablation and 1‐year recurrence rates in the AF ablation study cohort have been decreasing in part due to the improved catheter technology in the past 9 years. However, patients with a first recurrence continue to appear consistently even after 5 years from successful catheter ablation. Obesity,^22^ metabolic syndromes,^23^ and obstructive sleep apnea^24^ are associated with a high probability of an AF recurrence after catheter ablation. Therefore, strategies aiming at a reduction in AF development or progression, such as lifestyle and risk factor management, are necessary to improve the therapeutic outcome of AFCA.^25^, ^26^, ^27^, ^28^

### 4.3 Why persistent AF ablation is more likely affected by the AF duration?

In the present study, a longer AF duration, especially longer than 3 years, was associated with a higher clinical recurrence of atrial arrhythmias after catheter ablation in patients with PeAF, but not in subjects with PAF. There are several potential explanations for this outcome. First, PAF or PeAF without significant progression can be mostly manageable by a circumferential PV isolation.^21^ However, PeAF with significant remodeling is generally accompanied by multiple non‐PV triggers^29^ and appropriate treatment is limited to the current ablation technology. Second, the PeAF category includes an excessively broad spectrum of AF progression and remodeling compared with PAF. The definition of PeAF in the current guidelines^30^ is based only on a 1‐week sustained duration of AF and does not reflect the degree of atrial remodeling. Some of the early PeAF cases regressed to PAF after AAD therapy, however, several progressed to permanent AF. Third, the presence of subclinical AF may have delayed the ECG‐based diagnosis of AF.^12^ Therefore, in the case of asymptomatic or minimally symptomatic AF, the ECG‐based AF duration may be underestimated compared with the actual AF duration in patients with PeAF lasting more than 3 years.

### 4.4 Clinical implications

The recommended AF rhythm control in the current guidelines is intended for patients with symptomatic AF rather than ECG‐based AF.^30^, ^31^ However, AF progression to PeAF or permanent AF is accompanied by high cardiovascular risk and long‐term mortality.^32^, ^33^ In addition, metabolic syndrome, which is associated with AF progression, increases the nonthromboembolic adverse cardiac outcomes in patients with AF.^34^ Therefore, a symptom‐based AF ablation can be justified in the PAF state; however, when progressing to PeAF, rhythm control should be considered even in patients with minimal symptoms to prevent the progression to permanent AF. On the basis of the results from the current analysis, a favorable outcome of AFCA can be expected for patients with PAF regardless of the AF duration, or those with PeAF found within 3 years. When performing catheter ablation for patients with PeAF that has lasted more than 3 years, a higher recurrence rate should be carefully considered.

### 4.5 Limitations

The present study had several limitations. First, although we attempted to define the duration of AF in patients with a clear time point for the first ECG diagnosis, the possibility of a discrepancy between the ECG‐based AF duration and actual AF duration remains. Especially in patients with PAF, the duration of AF was more difficult to determine and thus the relationship between an AF recurrence and the AF duration in those patients was less clear. Second, contrary to the continuous rhythm monitoring using an implantable loop recorder, the current guideline‐based consistent rhythm monitoring schedule could not detect all the subclinical AF episodes. Third, this was an observational study from a single‐center cohort that included a highly selected group of patients referred for AFCA. Despite this limitation, a realistic and consistent rhythm monitoring method was applied for all patients included in the AF ablation study cohort based on real‐world practice.

## 5 CONCLUSION

A longer duration of AF was associated with a higher clinical recurrence rate after catheter ablation; however, a significance was only observed in patients with PeAF of more than 3 years. An AF duration‐dependent difference in the clinical recurrence rate was not observed in patients with PAF.

## Supporting information

Supporting information
