# Supplementary material for: Persistent atrial fibrillation over 3 years is associated with higher recurrence after catheter ablation
Source: J Cardiovasc Electrophysiol. 2020 Jan 15;31(2):457–64. doi: 10.1111/jce.14345 (PMC7027787; doi:10.1111/jce.14345)
Supplement: Supplementary file 1 — Supporting information [file JCE-31-457-s001.DOCX]

**Supplementary Table 1.** Sensitivity and specificity for predicting clinical recurrence of AF after AFCA on the basis of different cutoff ranges of AF duration (overall population, n=1,005).

| **Cutoff range of AF duration (year)** | **Sensitivity** | **Specificity** | **Youden’s index*** |
| --- | --- | --- | --- |
| ≥ 0.5 | 0.294 | 0.807 | 0.101 |
| ≥ 1.0 | 0.322 | 0.784 | 0.106 |
| ≥ 1.5 | 0.504 | 0.667 | 0.171 |
| ≥ 2.0 | 0.756 | 0.553 | 0.309 |
| ≥ 2.5 | 0.755 | 0.642 | 0.397 |
| **≥ 3.0** | **0.787** | **0.626** | **0.413** |
| ≥ 3.5 | 0.804 | 0.579 | 0.383 |
| ≥ 4.0 | 0.844 | 0.519 | 0.363 |
| ≥ 4.5 | 0.827 | 0.522 | 0.349 |
| ≥ 5.0 | 0.849 | 0.460 | 0.309 |
| ≥ 5.5 | 0.859 | 0.429 | 0.288 |
| ≥ 6.0 | 0.870 | 0.411 | 0.281 |
| ≥ 6.5 | 0.884 | 0.376 | 0.260 |
| ≥ 7.0 | 0.873 | 0.340 | 0.213 |
| ≥ 7.5 | 0.889 | 0.249 | 0.138 |

* Youden’s index = [ Sensitivity + Specificity - 1 ]

**Supplementary Table 2.** Predictors of a clinical recurrence after catheter ablation of AF among a propensity score-matched population between patients with persistent and paroxysmal AF

|  | **Univariate analysis** | | **Multivariate analysis** | |
| --- | --- | --- | --- | --- |
|  | **HR (95% CI)** | ***p*-value** | **HR (95% CI)** | ***p*-value** |
| Paroxysmal AF (n=387) |  |  |  |  |
| Age (year) | 0.989 (0.959–1.021) | 0.499 | 0.970 (0.935-1.008) | 0.117 |
| Male sex | 0.470 (0.233–0.948) | 0.035* | 0.537 (0.172-1.677) | 0.284 |
| BSA (m^2^) | 0.131 (0.019–0.913) | 0.040* | 0.402 (0.015-10.490) | 0.584 |
| BMI (kg/m^2^) | 0.940 (0.836–1.056) | 0.295 |  |  |
| Heart failure | 0.722 (0.211–2.475) | 0.605 |  |  |
| Hypertension | 1.294 (0.646–2.593) | 0.467 |  |  |
| Diabetes mellitus | 0.696 (0.236–2.057) | 0.513 |  |  |
| Stroke or TIA | 2.106 (0.929–4.775) | 0.075 |  |  |
| Vascular disease | 1.298 (0.510–3.300) | 0.584 |  |  |
| LA diameter (mm) | 0.958 (0.899–1.021) | 0.187 |  |  |
| LAVI (mL/m^2^) | 1.003 (0.975–1.032) | 0.820 |  |  |
| LV ejection fraction (%) | 1.014 (0.970–1.060) | 0.544 |  |  |
| E/Em | 0.939 (0.845–1.043) | 0.238 |  |  |
| LA voltage (mV) | 0.386 (0.173–0.862) | 0.020* | 0.455 (0.191-1.086) | 0.076 |
| AF duration (year) | 0.923 (0.815–1.046) | 0.208 | 0.983 (0.870-1.110) | 0.781 |
| Persistent AF (n=387) |  |  |  |  |
| Age (year) | 0.998 (0.982–1.014) | 0.780 | 0.992 (0.975-1.011) | 0.384 |
| Male sex | 1.043 (0.729–1.492) | 0.819 | 1.056 (0.631-1.759) | 0.842 |
| BSA (m^2^) | 1.508 (0.631–3.608) | 0.356 |  |  |
| BMI (kg/m^2^) | 1.032 (0.978–1.089) | 0.256 |  |  |
| Heart failure | 0.605 (0.319–1.148) | 0.124 |  |  |
| Hypertension | 0.884 (0.642–1.217) | 0.448 |  |  |
| Diabetes mellitus | 0.783 (0.495–1.240) | 0.297 |  |  |
| Stroke or TIA | 0.760 (0.471–1.227) | 0.262 |  |  |
| Vascular disease | 0.954 (0.603–1.510) | 0.840 |  |  |
| LA diameter (mm) | 1.016 (0.987–1.046) | 0.285 |  |  |
| LAVI (mL/m^2^) | 1.013 (1.000–1.026) | 0.050 |  |  |
| LV ejection fraction (%) | 1.008 (0.988–1.028) | 0.443 |  |  |
| E/Em | 0.978 (0.940–1.018) | 0.278 |  |  |
| LA voltage (mV) | 0.648 (0.418–1.006) | 0.053 |  |  |
| AF duration (year) | 1.438 (1.054–1.962) | 0.022* | 1.351 (1.037-1.812) | 0.034* |

HR, hazard ratio; CI, confidence interval; BSA, body surface area; BMI, body mass index; TIA, transient ischemic attack; LA, left atrium; LAVI, left atrial volume index; LV, left ventricle; E/Em, early mitral inflow velocity over the early diastolic mitral annular velocity; AF, atrial fibrillation. *p<0.05
